# Not All Street Food Is Bad: Low Prevalence of Antibiotic-Resistant *Salmonella enterica* in Ready-to-Eat (RTE) Meats in Ghana Is Associated with Good Vendors’ Knowledge of Meat Safety

**DOI:** 10.3390/foods10051011

**Published:** 2021-05-06

**Authors:** Martin Aduah, Frederick Adzitey, Daniel Gyamfi Amoako, Akebe Luther King Abia, Rejoice Ekli, Gabriel Ayum Teye, Amir H. M. Shariff, Nurul Huda

**Affiliations:** 1 Department of Animal Science, University for Development Studies, Tamale P.O. Box TL 1882, Ghana; kabahaduah@gmail.com (M.A.); rejoiceekli7@gmail.com (R.E.); teye.gabriel@yahoo.com (G.A.T.); 2 Department of Veterinary Science, University for Development Studies, Tamale P.O. Box TL 1882, Ghana; 3 Antimicrobial Research Unit, College of Health Sciences, University of KwaZulu-Natal, Durban 4001, South Africa; amoakodg@gmail.com (D.G.A.); lutherkinga@yahoo.fr (A.L.K.A.); 4 Faculty of Food Science and Nutrition, Universiti Malaysia Sabah, Jalan UMS, Kota Kinabalu 88400, Sabah, Malaysia; amir.husni@ums.edu.my; 5 Department of Food Science, Faculty of Agriculture, Universitas Sebalas Maret, Surakarta 57126, Central Java, Indonesia

**Keywords:** knowledge and practice, meat safety, *Salmonella enterica*, ready-to-eat meats, antibiotic resistance, Ghana, street vended food

## Abstract

Foodborne infections due to the consumption of meat is a significant threat to public health. However, good vendor and consumer knowledge of meat safety could prevent meat contamination with and transmission of foodborne pathogens like Salmonella. Thus, this study investigated the vendor and consumer perception, knowledge, and practices of meat safety regarding ready-to-eat (RTE) meat and how this affected the prevalence and antibiotic susceptibility of *Salmonella enterica* in RTE meats in the streets of Ghana. A semi-structured questionnaire was used to obtain the demographics, knowledge, and practices of meat safety data from RTE meat vendors (*n* = 300) and consumers (*n* = 382). *Salmonella enterica* detection was done according to the United State of America (USA)-Food and Drugs Administration (FDA) Bacteriological Analytical Manual. The disk diffusion method was used for antibiotic resistance testing. The results revealed that most of the respondents had heard of meat safety (98.3% vendors, 91.8% consumers) and knew that meat could be contaminated by poor handling (100.0% vendors, 88.9% consumers). The respondents knew that regular hand washing reduced the risk of meat contamination (100.0% vendors, 94.0% consumers). Responses to the practices of meat safety by vendors were generally better. A very low *Salmonella enterica* prevalence was observed in the samples, ranging between 0.0 and 4.0% for guinea fowl and beef, respectively. However, the six isolates obtained were resistant to five of the nine antibiotics tested, with all isolates displaying different resistance profiles. Overall, the good knowledge and practice of meat safety demonstrated by the respondents corroborated the negligible prevalence of *Salmonella* in this study, reiterating the importance of vendor meat safety knowledge. However, the presence of resistant *Salmonella enterica* in some of the meat samples, albeit in a very low prevalence, warrants stricter sanitary measures and greater meat safety awareness in the general population to prevent meat-borne infections and potential transmission of drug-resistant bacteria to humans.

## 1. Introduction

Meat is consumed by many people worldwide, probably because of its good taste and nutritive value. It has a high biological value and is easily absorbed and incorporated into human body proteins [1]. However, it also serves as a suitable medium for bacterial growth and is a major contributor to foodborne diseases [2,3]. The predominant foodborne bacterial species that have frequently been associated with meat include *Salmonella enterica, Escherichia coli*, *Campylobacter species, Clostridium species, Staphylococcus aureus, Listeria monocytogenes, Bacillus cereus, Shigella* spp., *Vibro parahaemolyticus*, and *Yersinia* spp. [4,5,6,7,8]. These organisms are linked to several human illnesses and deaths annually [5,9,10,11,12]. The World Health Organization estimated that 600 million people fall ill after eating contaminated food, and 420,000 die every year as a result [11]. Additionally, USD 110 billion is lost each year in productivity and medical expenses due to the consumption of unsafe foods.

The European Food Safety Authority and European Center for Disease Prevention and Control [5] indicated that out of 5079 food/waterborne outbreaks, *Salmonella* was the most common bacterium detected. Furthermore, *Salmonella* species from meat, meat products, and eggs were the highest risk source. A review by Omer et al. [12] on bacterial foodborne outbreaks related to red meat and meat products between 1980 and 2015 showed that *Salmonella* species caused 21 outbreaks, mostly in Europe and the United States of America. *Salmonellae* are responsible for millions of cases of enteric diseases, thousands of hospitalizations, and deaths worldwide each year [5,12]. Ninety-six (96) *Salmonella* outbreaks associated with beef were reported by Laufer et al. [13] in the United States.

The intensive use of clinically relevant antimicrobials in human and veterinary medicine for different purposes such as therapeutics, prophylactics, and growth promotion has increased the emergence and wide spread of antibiotic-resistant foodborne bacteria [14]. Foods contaminated with antibiotic-resistant bacteria are a major challenge to public health and have a deleterious impact on public health interventions [15]. Epidemiological data associated with the incidence of *Salmonella* and its antimicrobial resistance pattern is essential to develop an efficient mechanism toward its control at every level of the food processing and production chain to ensure food safety and public health [16].

Rane [17] indicated that street-vended foods are usually associated with foodborne diseases. Street ready-to-eat (RTE) meat vending is popular in the Bolgatanga Municipality of Ghana. The trade makes a significant contribution to the protein intake of the habitants of Ghana. However, the knowledge of RTE meat vendors/consumers on meat safety and how this could affect the level of microbial contamination of street-vended food have not been well established. There is also limited information on whether RTE meats produced in Ghana are contaminated by bacteria or contain resistant bacterial species. This paucity of information has created the overall perception that all street vended RTE meat is not safe for consumption, causing significant economic loss for people who make a living from this trade. Therefore, to sustain the livelihood of RTE meat vendors, while ensuring the health safety of consumers, this study investigated vendor and consumer perception, knowledge, and practices of meat safety regarding ready-to-eat (RTE) meat and how this affected the prevalence and antibiotic susceptibility of *Salmonella enterica* in RTE meats on the streets of Ghana.

## 2. Materials and Methods

### 2.1. Study Area

The study was conducted in Bolgatanga, the regional capital of the Upper East Region of Ghana. It is bordered to the north by the Bongo District, south and east by the Talensi and Nabdam Districts, and to the west by the Kassena/Nankana Municipality. Bolgatanga lies between latitudes 10°30′ and 10°50′ N and longitudes 0°30′ and 1°00′ W [18]. It is entirely urban and has a population of 66,685 [18].

### 2.2. Study Design, Population, and Questionnaire Administration

A descriptive survey was conducted using a semi-structured questionnaire (Supplementary Materials) to obtain information from grilled ready-to-eat (RTE) meat vendors and consumers on their knowledge and hygienic meat safety practices. Simple random sampling was used to select RTE meat vendors and consumers. All RTE meat vendors (*n* = 300) identified in the Bolgatanga Municipality were interviewed. For RTE meat consumers, the sample size was obtained by querying the population of Bolgatanga in the Sample Size Calculator [19]; and at a confident level of 95%, a sample size of 382 was obtained. The questionnaires used were developed according to comprehensive food safety literature reviews and divided into three main sections: demographic characteristics, knowledge on meat safety, and hygiene practices (Supplementary Materials).

### 2.3. Microbial Load

The microbial load was done according to Maturin and Peeler [20]. Briefly, an area of 10 cm^2^ of RTE (from approximately 25 g of a meat portion) beef, chevon, chicken, guinea fowl, mutton, or pork was swabbed. The swab was added to 9 mL of 0.1% buffered peptone water and homogenized for 2 min. Subsequently, decimal serial dilutions from 10^−1^ to 10^−4^ were made and plated on duplicate plate count agar. The plate count agar was incubated aerobically at 37 °C for 24 h. All media used were purchased from Oxoid Limited, Basingstoke, UK.

### 2.4. Isolation of Salmonella Enterica

A total of 300 RTE meat swab samples made up of beef (*n* = 50), chevon (*n* = 50), chicken (*n* = 50), guinea fowl (*n* = 50), mutton (*n* = 50), and pork (*n* = 50) were randomly collected aseptically from RTE meat vendors in the Bolgatanga Municipality from January to December 2019 and examined for the presence of *Salmonella enterica*. The isolation of *Salmonella enterica* was done according to the USA-FDA Bacteriological Analytical Manual [21]. Swabs were pre-enriched in buffered peptone water and incubated aerobically at 37 °C for 24 h. Afterward, they were enriched in Rappaport-Vassiliadis (incubated at 42 °C for 24 h) and selenite cystine (incubated at 37 °C for 24 h) broths. Aliquots from Rappaport-Vassiliadis and selenite cystine broths were streaked on xylose lysine deoxycholate and brilliant green agar. *Salmonella enterica* were confirmed using Gram stain, triple sugar iron agar, lysine iron agar, and *Salmonella* latex agglutination test. All incubations were done aerobically, and media used were purchased from Oxoid Limited, Basingstoke, UK.

### 2.5. Antibiotic Resistance Test

The disc diffusion method of Bauer et al. [22] was used for the antibiotic-resistant test. The *Salmonella enterica* isolates were examined against amoxycillin/clavulanic acid 30 µg (AMC), azithromycin 15 µg (AZM), ceftriaxone 30 µg (CRO), chloramphenicol 30 µg (C), ciprofloxacin 5 µg (CIP), gentamycin 10 µg (CN), teicoplanin 30 µg (TEC), tetracycline 30 µg (TE), and sulfamethoxazole/trimethoprim (SXT) antibiotics. The isolates were grown in trypticase soy broth and incubated aerobically at 37 °C for 18 h, after which, it was adjusted to 0.5 McFarland solution and spread plated on Muller Hinton agar. Five or four antibiotic discs were placed on the surface of the Muller Hinton agar plates and incubated aerobically at 37 °C for 24 h. The inhibition zones were measured, and the results were interpreted as sensitive, intermediate, or resistant according to the Clinical and Laboratory Standards Institute guidelines [23]. All media and antibiotic discs were purchased from Oxoid Limited, Basingstoke, UK.

### 2.6. Statistical Analysis

Data collected were analyzed using the Statistical Package for Social Sciences version 20 (IBM, Armonk, NY, USA). The total aerobic plate count was log-transformed and analyzed using one-way ANOVA of GenStat 12.2 Release 12.1 (Copyright) 2009, and means were separated using standard error of means. Chi-Square test (χ2) was used to determine the relationships between some of the parameters, and significant differences were considered when *p* ≤ 0.05. Analyzed results were presented in frequencies and percentages in tables.

## 3. Results

### 3.1. Demographic Characteristics of Ready-to-Eat (RTE) Meat Vendors and Consumers

The demographic characteristics of the grilled ready-to-eat (RTE) meat vendors and consumers is shown in Table 1. The knowledge of RTE meat vendors and consumers in meat safety and contamination, and their responses to hygienic practices is shown in Table 2 and Table 3, respectively. The majority of the RTE meat vendors and consumers were males (97.7% and 71.7%), aged between 21–40 years (77.3% and 65.4%), and had basic education (73.7% and 54.2%), respectively. The gender of RTE meat vendors did not influence the type of RTE product sold (*X*^2^ = 9.490a; df = 5, *p* = 0.091) and hearing of meat safety (*X*^2^ = 0.121a; df = 1, *p* = 0.727), but not the source of meat for sale (*X*^2^ = 24.474a; df = 2, *p* = 0.000). The gender of RTE meat consumers had an influence on the type of RTE meat preferred (*X*^2^ = 28.365a, df = 4, *p* = 0.00), but not hearing of meat safety (*X*^2^ = 0.861a, df = 3, *p* = 0.835) and knowing that meat can be contaminated by poor handling (*X*^2^ = 1.936a, df = 2, *p* = 0.380). The age of RTE meat vendors influenced the type of RTE meat sold (*X^2^* = 60.770a, df = 10, *p* = 0.000), but not hearing of meat safety (*X*^2^ = 0.049a, df = 2, *p* = 0.976) and the source of meat for sale (*X*^2^ = 9.448a, df = 4, *p* = 0.051). The age of RTE meat consumers influenced the type of RTE product preferred (*X*^2^ = 31.389a; df = 12 and *p* = 0.002), but not hearing of meat safety (*X*^2^ = 2.472a; df = 9, *p* = 0.982) and knowing that meat can be contaminated by poor handling (*X*^2^ = 3.327a, df = 6, *p* = 0.767). The educational level of RTE meat vendors did not influence the type of RTE meat preferred (*X*^2^ = 0.698a, df = 2, *p* = 0.705), hearing of meat safety (*X*^2^ = 0.698a, df = 2, *p* = 0.705), and knowing that meat can be contaminated by poor handling (*X*^2^ = 5.405a, df = 4, *p* = 0.248). The educational level of RTE meat consumers influenced the type of RTE meat preferred (*X*^2^ = 79.351a, df = 16, *p* = 0.000) and hearing of meat safety (*X*^2^ = 50.123a, df = 12, and *p* = 0.000), but not knowing that meat can be contaminated by poor handling (*X*^2^ = 9.022a, df = 8, *p* = 0.340).

### 3.2. Microbial Load and Prevalence of Salmonella Enterica in RTE Meats

The microbial load and prevalence of *Salmonella enterica* in the RTE meat samples is shown in Table 4. The microbial load was 4.17, 4.85, 4.02, 4.06, 2.53, and 3.37 log CFU/cm^2^ for mutton, chevon, pork, guinea fowl, chicken, and beef, respectively. RTE mutton (2.00%), chevon (2.00%), pork (2.00%), guinea fowl (4.00%), and chicken (2.00%) were positive for *Salmonella enterica*. *Salmonella enterica* was not detected in RTE beef (0.00%).

### 3.3. Prevalence and Antibiotic Resistance of Salmonella enterica Isolated from RTE Meats

A low prevalence of *Salmonella enterica* was observed in the RTE meat samples RTE mutton (2.00%), chevon (2.00%), pork (2.00%), guinea fowl (4.00%), and chicken (2.00%) were positive for *Salmonella enterica*. *Salmonella enterica* was not detected in RTE beef (0.00%).

**Figure 1:**
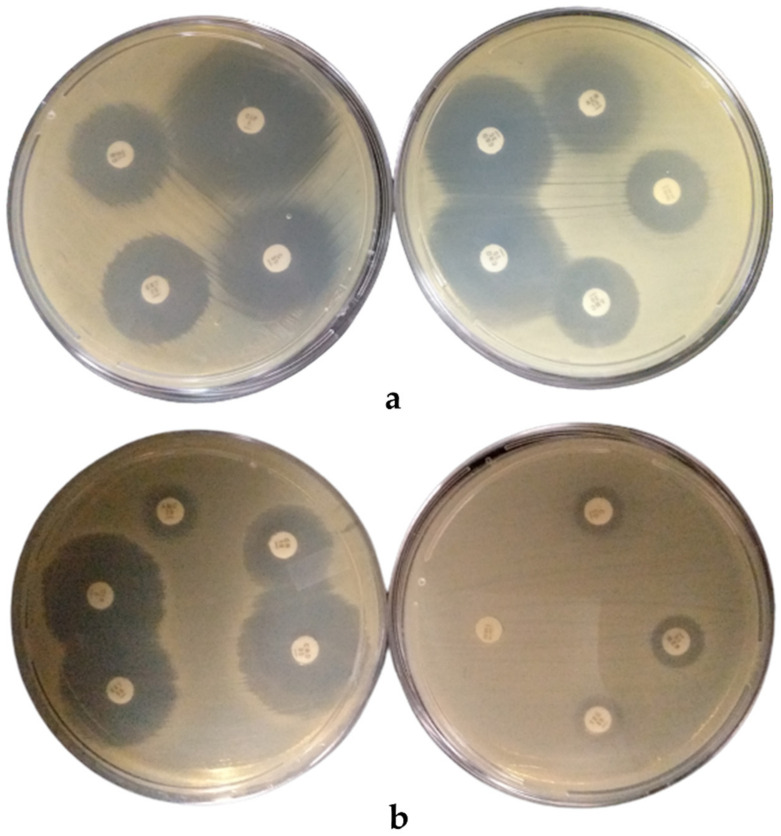
Plates with zones of inhibition (clear zones around each antibiotic disc) for (**a**) the control strain and (**b**) isolate S7.

The *Salmonella enterica* isolates showed resistance to amoxycillin/clavulanic acid, azithromycin, teicoplanin, and tetracycline, but susceptible to ceftriaxone, chloramphenicol, ciprofloxacin, and sulfamethoxazole/trimethoprim (Table 5 and Figure 1).

The antibiotic resistance profile and multiple antibiotic resistance (MAR) index of individual *Salmonella enterica* isolated from the RTE meats are shown in Table 6. Six different resistant profiles were observed. Multidrug resistance (resistant to three different antibiotics) was observed in 4/6 isolates, with MAR index ranging from 0.22 to 0.56.

## 4. Discussion

The observance of meat safety is essential to reduce the incidence of foodborne diseases associated with contaminated meats. This study showed that meat vendors and consumers had a relatively good knowledge of meat safety, which was reflected in the low prevalence of *Salmonella* in the samples analyzed.

Most of the grilled ready-to-eat (RTE) meat vendors and consumers were males, youth, and had their education up to the primary level. Similarly, it has been reported that the meat selling business is dominated by males, young people, and people with a low level of education [24,25,26,27,28].

The gender and age of the RTE meat vendors influenced where they bought meat for sale and the type of RTE meat sold, respectively. Most RTE meat vendors bought their meat from the abattoir and sold RTE pork, mutton, guinea fowl, chevon, beef, and chicken. The majority of the RTE meat vendors had received training in meat safety. This was reflected in their knowledge of meat safety since they were aware that meat could be contaminated by poor handling, and regular handwashing could reduce the risk of contamination. The majority also knew it was necessary to take leave from work when infected with a skin disease, refrigerate leftover meat, and that eating and drinking while selling meat increased the risk of meat contamination. It is worth noting that apart from Ghanaians (who formed the majority), other nationalities such as Burkinabe’s, Malians, and Nigeriens were also involved in the RTE meat vending business. Most of the RTE meat vendors owned one shop, had between 1–10 years of working experience, sold RTE meats based on consumer preference, and full-time basis. Adzitey et al. [28] reported that most meat sellers had heard about meat safety, but had no training in meat safety. Additionally, they obtained their meat from the abattoir, knew that eating contaminated meat could cause meat-borne disease, and took leave from work when infected with any disease.

The gender, age, and education of RTE meat consumers influenced the type of RTE meat preferred. The majority preferred RTE guinea fowl due to its good taste. Besides taste, the cost, safety, availability, and healthiness of the meat type contributed to the choice for a particular RTE meat. The educational level of RTE meat consumers influenced awareness of meat safety. Most RTE consumers had heard about meat safety and knew that meat could be contaminated by poor handling and reduced when vendors washed their hands regularly. The RTE meat consumers often consumed RTE meat once a week, mostly when they went out with friends. Adesokan and Raji [24] reported that meat handlers had good knowledge of safe meat handling. They also indicated that elderly meat handlers with higher educational levels and many years of working experience were more likely to understand better meat safety than younger ones with non-formal education and less working experience. Sulleyman et al. [27] found that most meat sellers had information on meat safety from relevant stakeholders, knew that eating contaminated meat could cause foodborne illness, but did not know the type of illness caused by eating contaminated meat.

The majority of RTE meat vendors obtained their meat from the abattoir due to its safety and quality. They also wash their cutting tables at the beginning and the end of work and disinfect their shops twice a week with isopropyl alcohol. They also sterilize their cutting tools and other equipment daily. Most RTE meat vendors wore aprons, which they washed daily, wore gloves sometimes, and did not smoke. Tegegne and Phyo [25] observed that all meat handlers knew about proper meat handling and hand washing, but this did not translate into strict food hygiene practices. A study by Adesokan and Raji [24] revealed that age, gender, education, and work experience were significantly associated with the level of safe meat handling by meat handlers. Meat handlers from private processing plants had better practices than those from government processing plants [24]. Adzitey et al. [26] reported that some meat sellers wore an apron while selling meat, and most of them washed their cutting tables and knives at the beginning and end of work each day.

This study revealed the presence of microbes in the RTE meats examined. Microbial contamination was highest for RTE chevon, mutton, guinea fowl, and pork, followed by RTE beef and chicken. RTE meats in Ghana are prepared using adequate heat, which can destroy all microbes. However, cross-contamination is possible due to improper personal hygiene and handling after preparation. The Ghana Standard Authority [29] recommends that microbial contamination for grilled meat should be <5 log cfu/g. This implies that all the RTE meats met the acceptable limit set by the Ghana Standard Authority. The microbial load observed in this study is also satisfactory [30,31]. Ampaw [31] reported microbial contamination of 4.732–7.267 log CFU/g in kebabs vended on the streets of Accra, Ghana. Agbodaze et al. [32] reported microbial contamination of 5.02 log cfu/g for grilled RTE meat samples bought from Osu, Nima, and Accra Central and attributed it to poor hygiene and sanitary measures adapted by RTE meat vendors. Therefore, the lower microbial load recorded in the current study indicates the impact of the vendors’ excellent meat safety knowledge on meat quality.

*Salmonella enterica* was detected in 2.0% (1 isolate each from RTE mutton, chevon, pork, and chicken, and two isolates from RTE guinea fowls) of the RTE meat samples examined. According to the Center for Food Safety [30], *Salmonella* should not be detected in 25 g of RTE meat samples; therefore, the six RTE meat samples in the present work can be described as unsatisfactory. This contamination occurred due to cross-contamination from faulty handling, especially after grilling the RTE meats. However, 98% of the RTE meats in this study were satisfactory and safe to eat regarding *Salmonella* contamination and subsequent infection. This study was in accordance with previous studies [33,34,35,36]. Terentjeva et al. [36] did not detect *Salmonella* in RTE meat samples examined in Latvia. *Salmonella* was detected in 1.5% RTE frozen chicken croquettes from Spain [34], 1.1% RTE Turkey meat products from the USA [33], and 0.64% RTE meat products from China [35]. In raw or fresh meat, Thai et al. [37] found that pork (39.6%) and chicken (42.9%) in Vietnam were contaminated with *Salmonella* species. Khaitsa et al. [33] reported a prevalence of 4.1% for *Salmonella* species in raw turkey collected from retail outlets in the USA. In South Africa, Mokgoph et al. [38] observed that 64.9% of chickens were positive for *Salmonella* species. *Salmonella* species were detected in 1.5% of minced meat and meat preparations, but were not detected in frozen meat (0.0%) in Latvia [36]. A study conducted in Serbia found no *Salmonella* species in RTE meats, albeit found them in meat preparations (7.0%) and minced beef (18.0%) [39]. Other Gram-negative bacteria such as *E. coli*, *Klebsiella* species, *Campylobacter* species, *Pseudomonas* species, and *Proteus* species have been reported in RTE meats [40,41,42]. The antibiotic resistance results indicated resistance to amoxicillin/clavulanic acid, azithromycin, and teicoplanin. The development of antibiotic resistance by bacteria has been linked to their misuse in animal production and treatment of animals [13,14,36]. This implies that the antibiotic resistance observed in the current study could be due to such use in animal production in the study area, and proper cooking is advised to eliminate any potential bacterial contamination. The resistance could also be from human contamination, which may be linked to the smaller percentage of respondents that did not observe proper meat safety measure. This finding suggests that food safety measures need to be strictly implemented, as even minute acts of negligence could result in meat contamination. Several studies conducted elsewhere have previously reported a high percentage of resistance in *Salmonella* isolated from different meat sources. For example, Terentjeva et al. [36] reported that *Salmonella* isolates from meat and meat products were resistant to sulfamethoxazole (40.0%), ciprofloxacin (25.0%), and tetracycline (20.0%), but were susceptible to azithromycin (100.0%). Comparatively, this study found a higher resistance to azithromycin and tetracycline, but not ciprofloxacin and sulfamethoxazole/trimethoprim. Similar to this study’s results, Harakeh et al. [43] found that *Salmonella species* isolated from meat-based fast foods showed 100.0% resistance to teicoplanin. According to Khaitsa et al. [33], 86.0% of *Salmonella* isolates from RTE meats exhibited multidrug resistance. According to Terentjeva et al. [36], 62.0% of *Salmonella* species isolated from meat and meat products in Latvia were resistant to at least one antimicrobial agent. Additionally, 25.0% the isolates were resistant to ciprofloxacin, 20.0% to tetracycline, and 0.0% to azithromycin [36]. This study found lower resistance to ciprofloxacin, but not tetracycline and azithromycin. In South Africa, *Salmonella* species from chicken carcass swabs were resistant to amoxycillin/clavulanic acid (3.7%), chloramphenicol (0.0%), ciprofloxacin (0.0%), gentamycin (0.0%), and sulfamethoxazole/trimethoprim (0.0%), while those from carcass drips were resistant to amoxycillin/clavulanic acid (7.4%), chloramphenicol (0.0%), ciprofloxacin (1.8%), gentamycin (5.8%), and sulfamethoxazole/trimethoprim (8.9%) [38]. Similar observations were made in this study except for the results for amoxycillin/clavulanic acid and gentamicin, which were higher in this study. Additionally, the *Salmonella* isolates exhibited resistance to one or more antibiotics, and the frequency varied among the type of samples (carcass swabs, cloacal swabs, and carcass drips) analyzed [38]. This concords with the present study. *Salmonella* species of chicken and pork origin from Vietnam were resistant to tetracycline (58.5%) and chloramphenicol (37.3%) [37]. Lower resistance to tetracycline and chloramphenicol occurred in this study. In Chile, *Salmonella* infantis from chicken meat was resistant to amoxycillin/clavulanic acid (3.5%), azithromycin (2.3%), ceftriaxone (67.8%), chloramphenicol (64.4%), ciprofloxacin (2.3%), gentamycin (11.5%), tetracycline (95.4%), and sulfamethoxazole/trimethoprim (49.4%) [44]. This study found higher resistance to amoxycillin/clavulanic acid, azithromycin, and gentamycin, but lower for the rest of the antibiotics tested. *Salmonella* species from meat preparations and minced beef were resistant to tetracycline (72.0%), gentamicin (8.0%), and sulfamethoxazole/trimethoprim (0.0%) [39]. The results for sulfamethoxazole/trimethoprim were similar to that of this study. However, tetracycline differed, with this study showing lower tetracycline and higher gentamicin resistance. While the antibiotic resistance percentages of this study may seem alarming, it should be noted that 98% of the samples analyzed in the current study were negative for *Salmonella*. Nevertheless, it is necessary to establish mechanisms to better communicate meat safety measures to meat vendors and the general population.

## 5. Conclusions

The ready-to-eat (RTE) meat vendors and consumers had relatively better knowledge of meat safety and observance of its practices. The microbial load on the RTE meats were within acceptable limits. Additionally, 98% of the samples analyzed were negative for *Salmonella,* and only six isolates were obtained from the remaining samples. Nevertheless, while the meat samples analyzed in this study were generally safe for consumption, regular surveillance of *Salmonella enterica* incidence in meat products, especially in grilled RTE, is essential and needed to ensure a continuous safe food supply. It is recommended that further research should characterize the *Salmonella enterica* isolates to determine their genetic relatedness and pathogenic potential. Furthermore, studies involving other municipalities and more female vendors would give a better picture of meat safety knowledge in the country and its impact on human health

## Figures and Tables

**Table 1:** Socio-demographic characteristics of ready-to-eat meat (RTE) vendors and consumers.

RTE Meat Vendors		RTE Meat Consumers	
Variables	Freq./Per. (%)	Variables	Freq./Per. (%)
Gender		Gender	
Male	293 (97.7)	Male	274 (71.7)
Female	7 (2.3)	Female	108 (28.3)
Age		Age	
Below 21 years	2 (0.7)	Below 21 years	35 (9.2)
21–40 years	231 (77.3)	21–40 years	250 (65.4)
41–60 years	66 (22.1)	41–60 years	87 (22.8)
Above 60 years	0 (0.0)	Above 60 years	10 (2.6)
Educational background		Educational background	
None	74 (24.7)	None	48 (12.6)
Basic	221 (73.7)	Basic	207 (54.2)
Secondary	5 (1.7)	Secondary	86 (22.5)
Tertiary	0 (0.0)	Tertiary	32 (8.4)
Others	0 (0.0)	Others	9 (2.4)
Nationality		How often do you consume RTE meat	
Ghanaian	265 (89.5)	Daily	20 (5.2)
Burkinabe	7 (2.4)	Once a month	114 (29.8)
Malian	16 (5.4)	2–3 times a week	29 (7.6)
Nigerien	8 (2.7)	Once a week	219 (57.3)
Years in business		What prompts you consume it	
Less than a year	0 (0.0)	My ‘’mouth sweet’’ me	22 (5.8)
1–5 years	131 (43.7)	When I go out with friends	332 (86.9)
6–10 years	130 (43.3)	For home consumption	27 (7.1)
Above 10 years	39 (13.0)	Others	1 (0.3)
Type of grilled RTE meat sold		Type of grilled RTE meat preferred	
Pork	50 (16.7)	Pork	32 (8.4)
Mutton	49 (16.3)	Mutton	22 (5.8)
Guinea fowl	51 (17.0)	Guinea fowl	266 (69.6)
Chevon	49 (16.3)	Chevon	43 (11.3)
Beef	51 (17.0)	Beef	19 (5.0)
Chicken	50 (16.7)	Reason for product preference	
Reason for product preference		Cheaper	6 (1.6)
Consumer preference	285 (95.0)	It is safe	2 (0.5)
Religion	9 (3.0)	Readily available	3 (0.8)
Cheaper	6 (2.0)	Has good taste	341 (89.3)
Occupational status		It is healthy	30 (7.9)
Full-time	281 (93.7)		
Part-time	19 (6.3)		
Alternative occupation if part-time			
Farming	22 (7.3)		
Number of shops			
One	290 (96.7)		
Two	10 (3.3)		

Freq. = frequency, Per. = percentage.

**Table 2:** Knowledge of ready-to-eat (RTE) meat vendors and consumers in meat safety and contamination.

RTE Meat Vendors			RTE Meat Consumers		
	Response			Response	
Variables	Yes: *n* (%)	No: *n* (%)	Variables	Yes: *n* (%)	No: *n* (%)
Have you ever heard of meat safety	295 (98.3)	5 (1.7)	Have you ever heard of meat safety	358 (91.8)	21 (5.5)
Do you know that meat can be contaminated by poor handling	300 (100)	0 (0.0)	Do you know that meat can be contaminated by poor handling	327 (88.9)	26 (7.1)
Knowledge on meat-borne diseases	289 (96.3)	6 (2.0)	Do you know that eating, drinking, and smoking by vendors while RTE meat increases the risk of contamination	70 (19.0)	282 (76.6)
Received training on meat safety	254 (85.5)	43 (14.5)	Do you know that regular washing of hands by vendors reduces the risk of contamination	359 (94.0)	23 (6.0)
Aware that eating, drinking, and smoking while selling meat increases the risk of meat contamination	297 (99.0)	3 (1.0)			
Aware that regular washing of hands reduces the risk of meat contamination	300 (100)	0 (0.0)			
Aware that using sterilized gloves reduces the risk of meat contamination	298 (99.3)	2 (0.7)			
Know that there is the need to take leave from work when infected with any disease	296 (98.7)	4 (1.3)			
Know that it is necessary to refrigerate leftover meat	284 (94.7)	16 (5.3)			

*n* = Number of respondents.

**Table 3:** Ready-to-eat meat vendor and consumer responses to hygienic practices.

Ready-to-Eat (RTE) Meat Vendors		Ready-to-Eat (RTE) Meat Consumers	
Variables	Freq./Per. (%)	Variables	Freq./Per. (%)
Source of meat for grilling		How should leftover grilled RTE meat be stored?	
Backyard slaughter	111 (37.0)	Refrigeration	256 (67.0)
Abattoir	182 (60.7)	Salting	8 (2.1)
Imported carcass	7 (2.3)	Smoking	105 (27.5)
Reasons for choice of source		Frying	13 (3.4)
Safe and quality	182 (60.7)		
Readily available	110 (36.7)	Where do you buy your grilled RTE meat?	
Cheap	8 (2.7)	Market	63 (16.5)
What do you sell meat on/in?		Roadside	208 (54.5)
An open table	7 (2.3)	Restaurant	35 (9.2)
Table with a net covering the meat	144 (48)	Drinking bar	76 (19.9)
Glass sieve	121 (40.3)		
Others	28 (9.3)	How is the RTE meat that you buy normally displayed?	
Frequency of washing cutting tables		On open table	58 (15.2)
At the beginning of work	20 (6.7)	Table with wire mesh covering	170 (44.5)
At the end of work	5 (1.7)	Glass sieve	147 (38.5)
At the beginning and at the end of work	275 (91.7)	Others	7 (1.8)
Do you disinfect your shop?			
Yes	298 (99.3)	Do you wash your hands before touching or eating RTE meat?	
No	2 (0.7)	Yes	93 (24.8)
How often do you disinfect your shop?		No	261 (69.6)
Once a week	128 (42.7)		
Twice a week	171 (57.0)	What do you use to wash if yes?	
Others	1 (0.3)	Only water	220 (73.3)
Type of disinfectant used		Soap and water	80 (26.7)
Isopropyl alcohol	284 (94.7)		
Iodine	12 (4.0)	Where do you eat your RTE meat?	
Hydrogen peroxide	4 (1.3)	On the street	47 (12.3)
Frequency of washing hands before touching meat		At home	85 (22.3)
Always	299 (99.7)	In a drinking bar	222 (58.1)
Sometimes	1 (0.3)	On the vendors’ table	28 (7.3)
Yes	300 (100.0)		
Only water	1 (0.3)		
Detergent and water	298 (99.3)		
Others	1 (0.3)		
Water	228 (76.0)		
Warm water	72 (24.0)		
Sterilization of cutting tools and other equipment			
Yes	283 (94.3)		
No	17 (5.7)		
Daily	161 (53.8)		
Twice a week	10 (3.3)		
Weekly	126 (42.1)		
Others	2 (0.7)		
Yes	294 (98.0)		
No	5 (1.7)		
Everyday	272 (91.3)		
Twice a week	18 (6.0)		
Once a week	8 (2.7)		
Always	9 (3.0)		
Sometimes	204 (68.0)		
Rarely	65 (21.7)		
Never	22 (7.3)		
Yes	5 (1.7)		
No	295 (98.3)		
Very dirty	1 (0.3)		
Dirty	4 (1.3)		
Clean	99 (33.0)		
Very clean	196 (65.3)		
Refrigeration	284 (94.7)		
Smoking	16 (5.3)		
Frying/Salting	0 (0.0)		

Freq. = frequency, Per. = percentage.

**Table 4:** Occurrence of *Salmonella enterica* and bacteria load in the ready-to-eat (RTE) meats.

RTE Meat Type	Total Sample Tested	No. (%) Positive	* Bacteria Load (log cfu/cm^2^
Mutton	50	1 (2.00)	4.17 ^bc^
Chevon	50	1 (2.00)	4.85 ^c^
Pork	50	1 (2.00)	4.02 ^bc^
Guinea Fowl	50	2 (4.00)	4.06 ^bc^
Chicken	50	1(2.00)	2.53 ^a^
Beef	50	0 (0.00)	3.37 ^ab^
Total/average	300	6 (2.00)	3.83

No. = number of samples positive for *Salmonella enterica.* * Standard error of difference = 0.294, ^abc^ Probability value = < 0.001.

**Table 5:** Antibiotic resistance of the *Salmonella enterica* isolated from ready-to-eat meats.

Antimicrobial	Resistant (%)	Susceptible (%)
Amoxycillin/Clavulanic acid 30 ug (AMC)	66.67	33.33
Azithromycin 15 ug (AZM)	83.33	16.67
Ceftriaxone 30 ug (CRO)	0.00	50.00
Chloramphenicol 30 ug (CHL)	0.00	83.33
Ciprofloxacin 5 ug (CIP)	0.00	83.33
Gentamycin 10 ug (GEN)	16.67	33.33
Teicoplanin 30 ug (TEC)	100.00	0.00
Tetracycline 30 ug (TET)	50.00	0.00
Sulfamethoxazole/Trimethoprim (SXT)	0.00	100.00
Overall	35.19	44.44

**Table 6:** Antibiotic resistance profile and multiple antibiotic resistance indexes of *Salmonella enterica.*

Codes	Meat Type	Number of Antibiotics	Antibiotic-Resistant Profile	MAR Index
S7	Mutton	5	AMC-AZM-TEC-TET-CHL	0.56
P3	Pork	2	AZM-TEC	0.22
Go44	Chevon	4	AZM-TEC-GEN-TET	0.44
G44	Guinea fowl	2	AMC-TEC	0.22
G49	Guinea fowl	3	AMC-AZM-TEC	0.33
C16	Chicken	4	AMC-AZM-TEC-TET	0.44

AMC = Amoxycillin/Clavulanic acid, AZM = Azithromycin, TEC = Teicoplanin, TET = Tetracycline, CHL = Chloramphenicol.

## Data Availability

All data have been included in the manuscript.

## Supplementary Materials

The following are available online at https://www.mdpi.com/article/10.3390/foods10051011/s1, Questionnaire for ready-to-eat (RTE) meat vendors.
