# Supplementary material for: Not All Street Food Is Bad: Low Prevalence of Antibiotic-Resistant Salmonella enterica in Ready-to-Eat (RTE) Meats in Ghana Is Associated with Good Vendors’ Knowledge of Meat Safety
Source: Foods. 2021 May 6;10(5):1011. doi: 10.3390/foods10051011 (PMC8148193; doi:10.3390/foods10051011)
Supplement: Supplementary file 1 [file foods-10-01011-s001.zip › foods-1163031-supplementary.pdf]

Questionnaire for ready-to-eat (RTE) meat vendors

University for Development Studies; Faculty of Agriculture

Animal Science Department

This study seeks to evaluate the knowledge and practices of meat safety by ready-to-eat (RTE) meat vendors on the streets of the Bolgatanga Municipality, Ghana.

Please, information given will be treated with high level of confidentiality and used only for academic purposes.

Tick where appropriate or state accordingly [√].

PART I: PERSONAL PROFILE

1. Gender: Male [ ] Female [ ]
2. Age (years): a. below 21 [ ] b. 21- 40 [ ] c. 41 - 60 [ ] d. above 60 [ ]
3. Nationality: a. Ghanaian b. Burkinabe c. Malian d. Niger e. Others (specify)…
4. Marital status: a. Married [ ] b. Single [ ] c. Divorced [ ] d. Others [ ]
5. Religion: a. Christianity [ ] b. Islamic [ ] c. Traditional [ ] d. Others [ ]
6. Educational background: a. None [ ] b. Basic [ ] c. Secondary [ ] d. Tertiary [ ]
   e. Others (specify)…………

PART II: OCCUPATIONAL PROFILE

6. How long have you been selling RTE meat?

a. Less than 1 year [ ] b. 1 -5 years [ ] c. 6-10 years [ ] d. above 10 years [ ]

7. Which of the following do you sell?

a. Pork [ ] b. Mutton [ ] c. Guinea fowl [ ] d. Chevon [ ] e. Chicken f. Beef g. Others [ ]

8. Why do you prefer selling the selected meat type?

a. Cheaper [ ] b. Consumer preference [ ] c. Religion [ ] d. Others (specify)….

9. Do you sell meat as a full or part time activity?

a. Fulltime [ ] b. Part-time [ ]

If part time, what work do you do?

10. How many meat shops do you have?

a. One [ ] b. Two [ ] c. More than three [ ]

PART III: KNOWLEDGE OF MEAT VENDORS ON MEAT SAFETY

11. Have you ever heard of meat safety?

a. Yes [ ] b. No [ ] If yes, where……………………………..

12. Do you know meat can be contaminated with bacteria/germs by poor handling?

a. Yes [ ] b. No [ ]

13. Do you also know that contaminated meat can cause meat borne diseases?

a. Yes [ ] b. No [ ]

If yes, give some examples of meat borne diseases you know………………….

14. Have you undergone any training course regarding safe meat handling?

a. Yes [ ] b. No [ ] If yes, where (specify): ………………………..

15. Are you affiliated to or a member of any meat sellers association?

a. Yes [ ] b. No [ ] If yes, please state the association: …………………

16. Are you aware that eating and drinking while selling meat increases the risk of meat contamination?

a. Yes [ ] b. No [ ]

17. Do you know that washing hands before and at regular intervals during the working day, especially after visiting the lavatory (toilet) reduces the risk of meat contamination?

a. Yes [ ] b. No [ ]

18. Are you aware that using sterilized gloves during work reduces the risk of meat contamination?

a. Yes [ ] b. No [ ]

19. Do you know it is necessary to take leave from work when infected by any disease?

a. Yes [ ] b. No [ ]

20. How do you preserve leftover RTE meat?

a. Refrigeration [ ] b. Salting [ ] c. Smoking d. Frying e. Others (specify)...

21. Why do you choose this method? a. Easily available b. Cheap c. Very effective

PART IV: MEAT SAFETY PRACTICES AMONG MEAT VENDORS

22. Where do you get your meat from?

a. Backyard slaughter [ ] b. Abattoir [ ] c. Imported carcass [ ] d. Others (specify):………………….

23. Briefly explain why you choose such sources?

a. Safe and quality b. Readily available c. Cheap d. Others (specify):……

24. Do you sell meat on/in?

a. An open table [ ] b. Table with a net covering the meat [ ] c. Glass sieve d. Others (specify):………..…

25. How often do you wash your chopping/cutting tables?

a. At the beginning of work [ ] b. At the end of work [ ] c. a and b c. Once a week [ ] d. Others (specify):……………………

26. Do you disinfect your meat shop?

a. Yes [ ] b. No [ ]

27. How often do you disinfect the shop?

a. Once a week [ ] b. Twice a week [ ] c. Once a month [ ] d. Others (specify)……

28. What disinfectant do you use?

a. Isopropyl alcohol, **[ ]** b**.** Iodine **[ ]** c. Hydrogen peroxide d. Pine-Sol e. Others

29. Do you wash your equipment used for selling meat?

a. Yes [ ] b. No [ ]

30. What do you use to rinse your equipment after washing?

a. Water [ ] b. Warm water [ ] c. Alcohol d. Others (specify)…………..

31. How often do you wash your hands before touching the RTE meat?

a. Always [ ] b. Sometimes [ ] c. Rarely [ ] d. Never [ ]

32. What do you use to wash your equipment?

a. Only water [ ] b. Detergent and water [ ] c. Others (specify)

……………

33. Do you ever sterilize your knives and other equipment?

a. Yes [ ] b. No [ ] If yes, how often

………………………….

34. Do you wear an apron during work?

a. Yes [ ] b. No [ ]

35. How often do you wash it, if yes?

a. Every day b. Twice a week c. Once a week d. Others

(specify)…………

36. Do you wear gloves during work?

a. Always [ ] b) Sometimes [ ] c) Rarely [ ] d) Never [

]

37. How often do you wash the gloves?

a. Every day b. Twice a week c. Once a week d. Others

(specify)…………

38. Do you smoke?

a. Yes [ ] b. No [ ] If yes, where do usually do

it?…………………

39. On a scale of 1 to 4, score meat venders on neatness of clothes.

1= very dirty [ ] 2= dirty [ ] 3= clean [ ] 4= very clean [ ]

(Interviewer should observe and provide an appropriate answer).

Table 1: Key for the assessment

Score	Description
Score of 1 or very dirty	Fresh and old meat particles/blood splashes found all over the front of the sellers' clothes.

Score of 2 or dirty	Half of the clothes covered with either fresh or old meat particles/blood splashes.
Score of 3 or clean	Less than quarter of the clothes is covered with meat, only fresh meat particles/blood splashes.
Score of 4 or very clean	When meat particles/blood splashes were not found on the sellers' clothes.

PART V: ATTITUDE OF RTE MEAT VENDORS TOWARDS MEAT SAFETY

On a scale of 1 to 3, state your readiness towards ensuring meat safety.

Use: 1= Agree

2= Uncertain

3= Disagree

40. Sell RTE meat in a neat enclosure.
41. Work area must be cleaned before start of work.
42. Wash my tables, knives and other equipment before start of work.
43. Hands should be washed before selling RTE meat.
44. Selling RTE meat with dirty hands should be avoided.
45. RTE meat should not be touched with wounded hand.
46. We should not rub our hands on face, hair, etc. while selling.
47. Jewelry (including wedding ring) and a watch can be worn while handling RTE meat.
48. Raw meat and RTE meat should not necessarily be separated.
49. Separate equipment must be used to handle raw meat and RTE meat.

50. We must cover our mouth and nose when coughing or sneezing.

51. Disinfecting my meat shop regularly.

52. Wear clean apron when selling.

53. Defrosted RTE meat should not be refrozen.

54. Clean apron can be used as a towel to clean hand.

55. The same towel can be used to clean many places in the meat shop.

56. Like to use clean gloves.

57. We should not smoke while selling RTE meat.

58. Be trained on meat safety issues.

59. Always refrigerate leftover RTE meat.

60. Adhere to food safety rules and regulations.

University for Development Studies; Faculty of Agriculture

Department of Animal Science

This study seeks to evaluate the knowledge and practices of meat safety by ready-to-eat (RTE) meat consumers on the streets of the Bolgatanga Municipality, Ghana.

Please, information given will be treated with high level of confidentiality and used only for academic purposes.

Tick where appropriate or state accordingly [√].

PART I: PERSONAL PROFILE

1. Gender: Male [ ] Female [ ]
2. Age (years): a. below 21 [ ] b. 21- 40 [ ] c. 41 - 60 [ ] d. above 60 [ ]
3. Marital status: a. Married [ ] b. Single [ ] c. Divorced [ ] d. In a relationship [ ] e. Others [ ]
4. Religion: a. Christianity [ ] b. Islamic [ ] c. Traditional [ ] d. Others [ ]
5. Educational background: a. None [ ] b. Basic [ ] c. Secondary [ ] d.Tertiary [ ]

   e. Others (specify)…………

6. Tribe: a. Frafra [ ] b. Bulisa [ ] c Kasena d. Manprusi e. Others (specify)……

PART II: PATTERN OF MEAT CONSUMPTION

7. Do you consume RTE meat? Yes [ ] No [ ]

8. If yes, which of the following RTE meat do you prefer? a. Pork [ ] b.

Mutton [ ] c. Guinea fowl [ ] d. Chevon [ ] e. Beef [ ]

9. What is your reason for your preference in 8 above? a. readily available [ ] b. has good taste [ ] c. it is healthy [ ] d. it is cheap [ ] e. It is safe [ ] f. (specify)………

10. How often do you consume RTE meat products? a) Daily [ ] b. Once a month [ ] c. 2-3 times a week [ ] d. Once a week [ ] e. Others (specify)……..

11. What prompts you to consume RTE Meat? a. my mouth sweet me [ ] b. when I go out with friend(s) in the evening[ ] c. for home consumption d. Others (specify)……

PART III: KNOWLEDGE OF MEAT CONSUMERS ON MEAT SAFETY

12. Have you ever heard of meat safety?

a. No [ ] b. Yes [ ]

13. If yes, by who or what means? a. Health officer [ ] b. Media [ ] c. Veterinary officer [ ] d. Teacher [ ] e. Others (specify)……

14. Do you know meat can be contaminated with bacteria/germs by poor handling during grilling and can cause meat-borne diseases? a.Yes[ ] b. No [ ]

If yes give examples of some of the meat-borne diseases………….

15. Are you aware that eating, drinking and smoking by vendors while selling RTE meat to you increases the risk of its contamination?

a. Yes [ ] b. No [ ]

16. Do you know that when the vendor washes hands before and at regular intervals during the working day, especially after visiting the lavatory (toilet) reduces the risk of meat contamination?

a. Yes [ ] b. No [ ]

17. In your opinion how should left-over RTE meat be preserved?

a. Refrigeration [ ] b. Salting [ ] c. Smoking d. Frying e. Others (specify)..

Why did you choose this method?...............

PART IV: MEAT SAFETY PRACTICES AMONG RTE MEAT CONSUMERS

18. Where do you buy RTE meat from?

a. Market [ ] b. Road side [ ] c. Restaurant [ ] d. Drinking bar [ ] e. Others (specify):……………

19. How is the RTE meat (in 18 above) normally displayed?

a. On an open table [ ] b. Table with a net covering the meat [ ]

c. Glass sieve d. Others (specify):………..…

20. Do you wash your hands before touching or eating RTE meat ?

a. Yes [ ] b. No [ ]

21. If yes what do you use a. only water, b. soap and water, c. only warm water d. soap and warm water e. Others (specify):……………

22. Where do you eat your RTE meat? a. On the street [ ] b. At home [ ]

c. In a drinking bar [ ] d. On the venders table [ ]

PART V: ATTITUDE OF RTE MEAT CONSUMERS TOWARDS MEAT SAFETY

On a scale of 1 to 3, state your readiness to ensure that you consume meat that is microbiologically safe.

Use: 1= Agree

2= Uncertain

3= Disagree

23. Buy RTE meat displayed in a neat enclosure where the work area and the equipment are cleaned before and the end of work respectively.

24. I will not buy RTE meat from a vendor who is coughing and sneezing

25. Vendors should not rub their hands on face, nose, hair, etc. while selling.

RTE meat

26. Raw meat and RTE meat should be separated with separate equipment for handling them.

27. Vendors must disinfect their meat shops regularly.

28. Vendors wear apron, gloves and mouth mask while selling meat

29. Jewelry (including wedding ring) and a watch can be worn while handling RTE meat.
